# 3’-O-Methylorobol Inhibits the Voltage-Gated Sodium Channel Nav1.7 with Anti-Itch Efficacy in A Histamine-Dependent Itch Mouse Model

**DOI:** 10.3390/ijms20236058

**Published:** 2019-12-01

**Authors:** Fan Zhang, Ying Wu, Shuwen Xue, Shuangyan Wang, Chunlei Zhang, Zhengyu Cao

**Affiliations:** State Key Laboratory of Natural Medicines and Jiangsu Provincial Key Laboratory for TCM Evaluation and Translational Development, School of Traditional Chinese Pharmacy, China Pharmaceutical University, Nanjing 211198, China; zhangfan20111112@126.com (F.Z.); wingwycpu@126.com (Y.W.); xsw19850856166@163.com (S.X.); shuangyanwcpu@163.com (S.W.)

**Keywords:** Nav1.7, 3’-O-methylorobol, gating modifier, itch relief, lead compound

## Abstract

An itch is a clinical complication that affects millions of patients. However, few treatment options are available. The voltage-gated sodium channel Nav1.7 is predominantly expressed in peripheral sensory neurons and is responsible for the rising phase of action potentials, thereby mediating nociceptive conduction. A gain-of-function mutation of Nav1.7 results in the hyperexcitability of sensory neurons and causes the inherited paroxysmal itch. Conversely, a monoclonal antibody that selectively inhibits Nav1.7 is able to effectively suppress the histamine-dependent itch in mice. Therefore, Nav1.7 inhibitors may possess the potential to relieve the itch. In the present study, using whole-cell voltage-clamp recordings, we demonstrated that 3’-O-methylorobol inhibited Na^+^ currents in Nav1.7-CHO cells and tetrodotoxin-sensitive Na^+^ currents in mouse dorsal root ganglion (DRG) neurons with IC_50_ (half-maximal inhibitory concentration) values of 3.46 and 6.60 μM, respectively. 3’-O-methylorobol also suppressed the tetrodotoxin-resistant Na^+^ currents in DRG neurons, though with reduced potency (~43% inhibition at 30 µM). 3’-O-methylorobol (10 µM) affected the Nav1.7 by shifting the half-maximal voltage (V_1/2_) of activation to a depolarizing direction by ~6.76 mV, and it shifted the V_1/2_ of inactivation to a hyperpolarizing direction by ~16.79 mV. An analysis of 3’-O-methylorobol activity toward an array of itch targets revealed that 3’-O-methylorobol was without effect on histamine H_1_ receptor, TRPV1, TRPV3, TRPV4, TRPC4 and TRPM8. The intrathecal administration of 3’-O-methylorobol significantly attenuated compound 48/80-induced histamine-dependent spontaneous scratching bouts and the expression level of *c-fos* in the nuclei of spinal dorsal horn neurons with a comparable efficacy to that of cyproheptadine. Our data illustrated the therapeutic potential for 3’-O-methylorobol for histamine-dependent itching, and the small molecule inhibition of Nav1.7 may represent a useful strategy to develop novel therapeutics for itching.

## 1. Introduction

An itch is an unpleasant sensation that induces the urge to seek out the source and scratch [1]. As one of the major complications of dermatological and other diseases, such as diabetic neuropathy, chronic kidney disease, postherpetic neuralgia, and some cancers, almost one-third of the global population experiences an itch in a given week [2,3]. Chronic and severe acute itches are difficult to alleviate because they are often associated with psychological disturbances such as depression or sleep deprivation and thus severely damage the quality of life [4]. Histamine, as the most well-known endogenous pruritic agent that is released by mast cells, keratinocytes and neurons, activates a subset of peripheral sensory neurons that transmits pruritic stimulus information to the brain to evoke itching [5,6]. Therefore, an itch can generally be classified as histamine-dependent or histamine-independent. In the past decade, substantial evidence has demonstrated that the capsaicin-activated ion channel TRPV1 and the cold-activated ion channel TRPM8 in peripheral neurons, as well as the transient receptor potential (TRP) cation channels TRPV3 and TRPV4 in epidermal keratinocytes, are the key regulators of histaminergic itching [7,8,9,10]. The pharmacological inhibition of TRPV1, TRPV3, TRPV4 or TRPM8 has been shown to effectively reduced scratching behavior in the mouse models of the histaminergic itch [8,10,11,12,13]. These findings indicate that ion channels are important targets in itch management.

The voltage-gated sodium channel (VGSC) subtype Nav1.7 is a transmembrane protein that is widely expressed in peripheral nociceptive neurons. Nav1.7 plays a crucial role in the initiation and conduction of action potentials through the peripheral nervous system [14]. Recently, it has been reported that the gain-of-function mutation of Nav1.7 (I739V) leads to the inherited paroxysmal itch by increasing the hyperexcitability of sensory neurons [15,16]. Conversely, a monoclonal antibody that suppresses Nav1.7 activity by interacting with the voltage sensor effectively inhibits the histamine-dependent itch in mice [17]. Recently, it was reported that the pharmacological blockade of Nav1.7 reduced itching induced by methylglyoxal or in streptozotocin-induced diabetic mice, indicating that Nav1.7 played a key role in itching in a mouse model of type 1 diabetes [18]. All of these significant findings suggest that Nav1.7 is a novel and promising target for itch management.

In the present study, we identified an isoflavonoid, 3’-O-methylorobol, as a Nav1.7 inhibitor from a plant-derived natural product library. We demonstrated that 3’-O-methylorobol potently inhibited Nav1.7 and tetrodotoxin-sensitive (TTX-S) VGSC currents in mice dorsal root ganglion (DRG) neurons with a much weaker effect on tetrodotoxin-resistant (TTX-R) Na^+^ currents. 3’-O-methylorobol showed no effect against an array of itch targets including the H_1_ receptor, TRPV1, TRPV3, TRPV4, TRPC4 and TRPM8. Moreover, we demonstrated that in a mouse model of a histamine-dependent itch, 3’-O-methylorobol attenuated the scratching bouts with a comparable efficacy to cyproheptadine. Our research indicates that 3’-O-methylorobol may be a useful lead compound for the development of novel antipruritics.

## 2. Results

### 2.1. Inhibitory Effects of 3’-O-Methylorobol on Nav1.7 Stably Expressed in CHO Cells

**Figure 1:**
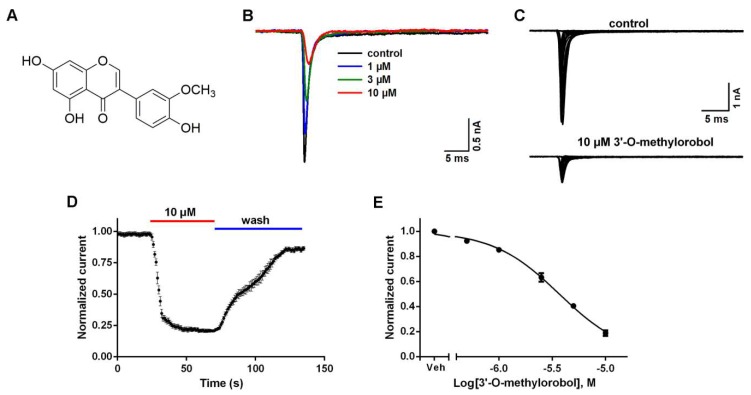
Effects of 3’-O-methylorobol on a Nav1.7 current stably expressed in CHO cells. (**A**) Chemical structure of 3’-O-methylorobol. (**B**) Representative traces of 3’-O-methylorobol suppression of Nav1.7 currents. The Nav1.7 current was evoked by a 50-ms depolarizing voltage of −20 mV from a holding potential of −80 mV. (**C**) Representative traces of Nav1.7 currents in the different depolarization potentials in the absence and presence of 10 µM of 3’-O-methylorobol. Currents were evoked by 50 ms depolarization voltages from −100 to 30 mV in steps of 5 mV. (**D**) Time–response relationship of the 3’-O-methylorobol suppression of Nav1.7 currents and the reversal of inhibition by washing with an external solution. (**E**) Concentration–inhibition relationship of 3’-O-methylorobol-suppressed Nav1.7 currents. Data points are shown as the mean ± SEM; *n* = 4–6.

To identify the potent inhibitors of Nav1.7, we screened a chemical library containing 576 plant-derived natural compounds by using whole-cell patch-clamp recording. As a result, 3’-O-methylorobol (C_16_H_12_O_6_, *m*/*z* = 300.2628), an isoflavonoid compound (Figure 1A), exhibited an inhibitory effect. The Nav1.7 current was triggered by a 50-ms depolarizing voltage of −20 mV from the clamped voltage of −80 mV in Nav1.7-CHO cells. 3’-O-methylorobol suppressed the Nav1.7 currents triggered by −20 mV and different depolarization potentials (Figure 1B,C). The time course for the 3’-O-methylorobol (10 μM) inhibition of Nav1.7 was rapid (τ_on_ = 19.3 ± 1.5 s), and the current displayed a relatively slow recovery (τ_off_ = 46 ± 3.3 s) by washing (Figure 1D). 3’-O-methylorobol concentration-dependently suppressed the Na^+^ currents in Nav1.7-CHO cells with an IC_50_ (half-maximal inhibitory concentration) value of 3.46 µM (95% confidence interval (95% CI): 2.17–5.69 µM) (Figure 1E).

### 2.2. Influences of 3’-O-Methylorobol on the Channel Kinetics of Nav1.7 Stably Expressed in CHO Cells

**Figure 2:**
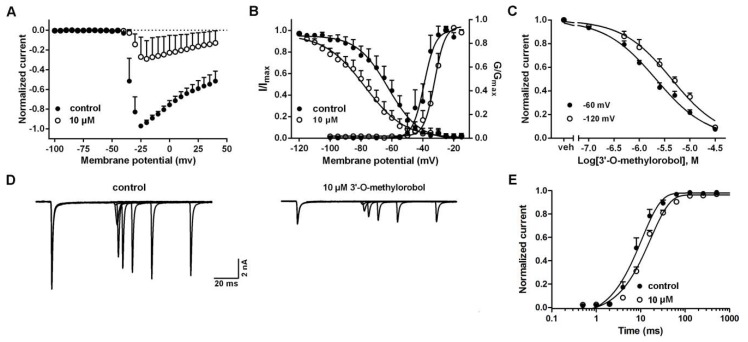
Influences of 3’-O-methylorobol on the channel kinetics of Nav1.7 stably expressed in CHO cells. (**A**) Normalized current–voltage (I–V) relationship for the Nav1.7 current before and after the application of 10 μM of 3’-O-methylorobol. (**B**) Effect of 10 μM of 3’-O-methylorobol on the steady-state activation and inactivation of Nav1.7. The steady-state inactivation was examined using a standard double-pulse protocol, in which a series of prepulses with potentials ranging from −130 to +20 mV with a 5 mV increment were applied for 500 ms before the Na^+^ current was triggered by a 50 ms depolarizing voltage to −20 mV; the clamped voltage of the cells was −100 mV. (**C**) Concentration–inhibition relationship of 3’-O-methylorobol on the Nav1.7 current at holding potentials of −120 and −60 mV. (**D**) Representative current traces of Nav1.7 currents before and after the application of 10 μM of 3’-O-methylorobol, indicating the rate of recovery from inactivation (repriming) at −80 mV. The cells were prepulsed to −15 mV for 20 ms to inactivate the Nav1.7 current and then returned to the recovery voltage (−80 mV) for increasing recovery durations prior to a test potential of −20 mV. (**E**) Recovery curves of the Nav1.7 current from fast inactivation in the absence and presence of 10 μM of 3’-O-methylorobol. Data points are shown as the mean ± SEM; *n* = 4–6.

Given the inhibition of the Nav1.7 current, the effects of 3’-O-methylorobol on the channel kinetics of Nav1.7 were examined. To test the effects of 3’-O-methylorobol on Nav1.7 activation, the Na^+^ currents were triggered by depolarized pulses from −100 to +40 mV in 5 mV steps in the absence or presence of 3’-O-methylorobol (10 µM) (Figure 2A). The current–voltage (I–V) relationships of Nav1.7 showed that 3’-O-methylorobol slightly shifted the active voltage of the peak current to a depolarization direction (5 mV) without affecting the initial activated voltage. The effects of 3’-O-methylorobol on the steady-state activation and inactivation of Nav1.7 were examined. After the application of 10 μM of 3’-O-methylorobol, the half-maximal voltage (V_1/2_) of the steady-state activation and inactivation were shifted from −39.18 ± 0.97 to −32.42 ± 0.57 mV (*n* = 5, *p* < 0.01) and from −63.09 ± 1.59 to −80.06 ± 2.12 mV (*n* = 5, *p* < 0.01), respectively (Figure 2B). We next investigated whether 3’-O-methylorobol preferentially interacted with the inactivated state of Nav1.7. As shown in Figure 2C, at test holding potentials of −120 and −60 mV, the IC_50_ values were 4.31 µM (3.59–5.14 µM, 95% CI) and 2.12 µM (1.86–2.42 µM, 95% CI), respectively. Furthermore, we analyzed the effect of 3’-O-methylorobol on the repriming kinetics (recovery from inactivation) of Nav1.7. Consistent with the alteration of the inactivation kinetics of Nav1.7, bath application of 3’-O-methylorobol (10 μM), the rate of recovery from inactivation slowed from 7.43 ± 0.25 to 11.78 ± 0.14 ms (*n* = 6, *p* < 0.01) (Figure 2D,E). Therefore, 3’-O-methylorobol was found to be a gating modifier compound of Nav1.7.

### 2.3. Effects of 3’-O-Methylorobol on TTX-S and TTX-R Na^+^ Currents in DRG Neurons

**Figure 3:**
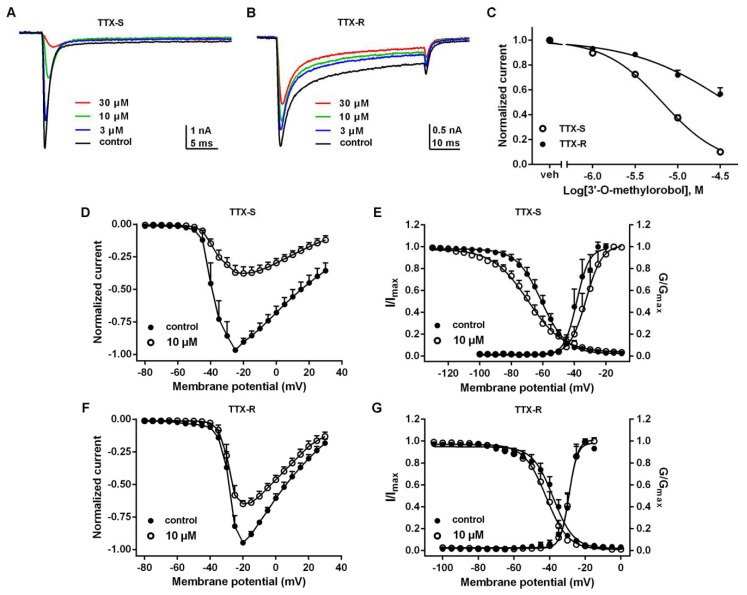
Effects of 3’-O-methylorobol on tetrodotoxin-sensitive (TTX-S) and tetrodotoxin-resistant (TTX-R) Na^+^ currents in dorsal root ganglion (DRG) neurons. (**A**) Representative traces of TTX-S Na^+^ currents before and after the application of different concentrations of 3’-O-methylorobol. TTX-S Na^+^ currents were triggered by a 50-ms step depolarization to −20 mV from a clamped voltage of −80 mV. (**B**) Representative traces of TTX-R Na^+^ currents before and after incubation with different concentrations of 3’-O-methylorobol. TTX-R Na^+^ currents were triggered by a 50-ms step depolarization to −15 mV from a clamped voltage of −80 mV. (**C**) Concentration–response relationships of the 3’-O-methylorobol inhibition of TTX-S and TTX-R Na^+^ currents. Normalized I–V relationship for (**D**) TTX-S Na^+^ currents and (**F**) TTX-R Na^+^ currents in the absence and presence of 10 μM of 3’-O-methylorobol. (**E**) Effect of 10 μM 3’-O-methylorobol on the steady-state activation and inactivation of TTX-S Na^+^ currents and (**G**)TTX-R Na^+^ currents. Data points are shown as the mean ± SEM; *n* = 4–10.

Nav1.7 is primarily expressed in peripheral nociceptive neurons and is the major contributor of TTX-S Na^+^ currents in DRG neurons. Therefore, we evaluated the effects of 3’-O-methylorobol on both TTX-S and TTX-R Na^+^ currents in DRG neurons. As shown in Figure 3A,B, 3’-O-methylorobol inhibited the TTX-S Na^+^ currents in the large diameter DRG neurons with an IC_50_ value of 6.6 (6.11–7.13 µM, 95% CI), with the maximal inhibition reaching 85% at a concentration of 30 μM (Figure 3C). 3’-O-methylorobol only moderately suppressed the TTX-R Na^+^ currents recorded in the small diameter DRG neurons, where the maximal inhibition was ~43% at a concentration of 30 μM (Figure 3A,C). The effects of 3’-O-methylorobol on the I–V relationships of TTX-S and TTX-R Na^+^ currents on DRG were examined. Upon application of 10 μM 3’-O-methylorobol, the initial activated voltage of both TTX-S and TTX-R Na^+^ currents were unchanged. 3’-O-methylorobol shifted the active voltage of the peak inward current of TTX-S Na^+^ currents to a depolarized direction (5 mV) while not effecting the active voltage of the peak current of TTX-R Na^+^ currents (Figure 3D,F). Furthermore, we examined the effects of 3’-O-methylorobol on the steady-state activation and inactivation of TTX-S and TTX-R Na^+^ currents. 3’-O-methylorobol (10 μM) shifted the half-activation voltage and half-inactivation voltage of TTX-S Na^+^ currents from −38.54 ± 1.01 to −32.50 ± 1.00 mV (*n* = 10, *p* < 0.01) and from −60.82 ± 0.60 to −69.38 ± 0.97 mV (*n* = 9, *p* < 0.01), respectively (Figure 3E). 3’-O-methylorobol at a concentration of 10 μM did not shift the steady-state inactivation of the TTX-R Na^+^ currents, but it did induce a weak hyperpolarization shift of the half-inactivation voltage from −37.95 ± 0.62 to −41.92 ± 0.57 mV (*n* = 6, *p* < 0.01) mV (Figure 3G). Therefore, 3’-O-methylorobol preferentially inhibited the TTX-S Na^+^ currents by modifying the kinetics of activation and inactivation of the channel.

Nav1.6 and Nav1.8 are also expressed in DRG neurons. We therefore investigated the effect of 3’-O-methylorobol on Nav1.6 and Nav1.8. The application of 3’-O-methylorobol (10 μM) suppressed both Nav1.6 and Nav1.8 currents with maximal inhibitions of 39% and 23%, respectively (Figure S1).

### 2.4. Effects of 3’-O-Methylorobol on Action Potentials Firing in Primary Cultured DRG Neurons

**Figure 4:**
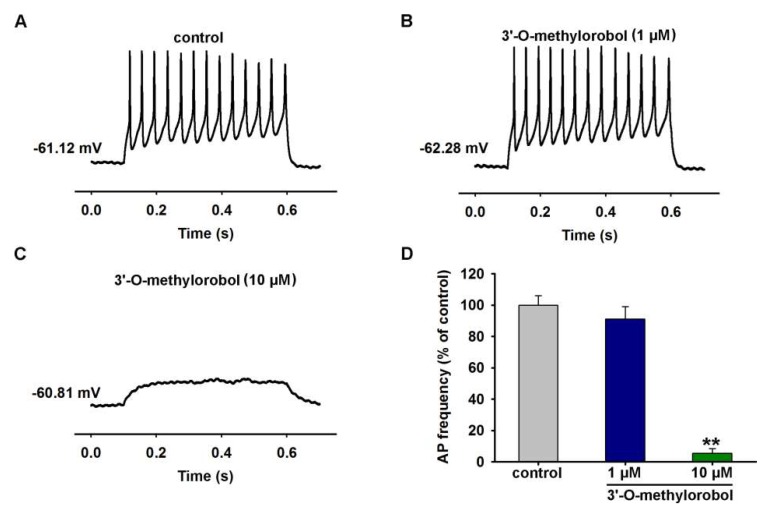
3’-O-methylorobol blocked current-evoked action potentials firing in primary cultured DRG neurons. (**A–C**) Representative traces of action potentials (APs) evoked by an injection of 100-pA current in DRG neurons in the absence and presence of different concentrations of 3’-O-methylorobol. (**D**) Quantification of the 3’-O-methylorobol suppression of 100-pA current-induced APs in acutely dissociated mice DRG neurons. Each data point represents the mean ± SEM; *n* = 5–7. ** *p* < 0.01, 3’-O-methylorobol vs. control.

Given the inhibition of the Na^+^ currents by 3’-O-methylorobol in DRG neurons, we examined the effect of 3’-O-methylorobol on the action potentials (APs) firing in primary-cultured DRG neurons. 3’-O-methylorobol at 1 μM slightly reduced the APs firing, while 3’-O-methylorobol abolished the APs firing at the concentration of 10 μM (Figure 4).

### 2.5. Effect of 3’-O-Methylorobol on Histamine-Dependent Itch Receptor and Ion Channels

**Figure 5:**
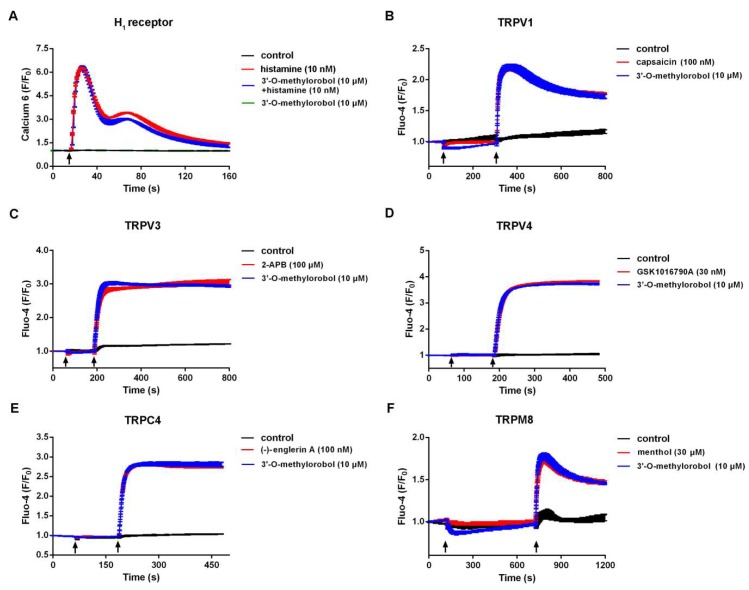
Effect of 3’-O-methylorobol on histamine-dependent itch receptor and ion channels. The effect of 3’-O-methylorobol (10 μM) on the H_1_ receptor (**A**), TRPV1 (**B**), TRPV3 (**C**), TRPV4 (**D**), TRPC4 (**E**), and TRPM8 (**F**) was evaluated by determination of intracellular Ca^2+^ concentration. The intracellular Ca^2+^ elevations induced by histamine (10 nM), capsaicin (100 nM, for TRPV1), 2-APB (100 μM, for TRPV3), GSK1016790A (30 nM, for TRPV4), (−)-Englerin A (100 nM, for TRPC4), and menthol (30 μM, for TRPM8), were recorded by a FLIPR^Tetra^. H_1_ receptors were stably expressed in CHO cells. For (**A**), the arrow indicates the addition of histamine and 3’-O-methylorobol to final concentrations as noted. For (**B**–**F**), the arrows indicate the addition of 3’-O-methylorobol and agonists for the first and second arrow, respectively. TRPV1, TRPV3, TRPV4, TRPC4, and TRPM8 were stably expressed in HEK293 cells. The experiments were performed twice, each in triplicate.

We next evaluated the effect of 3’-O-methylorobol on other itch-related targets, including the histamine H_1_ receptor, TRPV1, TRPV3, TRPV4, TRPC4, and TRPM8 in a heterologous expressing system, because these targets have been proven to play crucial roles in histamine-dependent itching. By using a fluorescent imaging plate reader (FLIPR^Tetra®^), the effect of 3’-O-methylorobol on agonists-induced intracellular Ca^2+^ change was determined. As shown in Figure 5A, 3’-O-methylorobol (10 µM) affected neither the basal Ca^2+^ level nor the histamine (10 nM)-induced Ca^2+^ influx. As for itch-related ion channels, the application of 3’-O-methylorobol (10 μM) had no effect on capsaicin (100 nM), 2-APB (100 μM), GSK1016790A (30 nM), (−)-Englerin A (100 nM), and menthol (30 μM)-induced intracellular Ca^2+^ elevation in HEK293 cells expressing TRPV1, TRPV3, TRPV4, TRPC4, and TRPM8, respectively (Figure 5B–F). Together, these data excluded the H_1_ receptor, TRPV1, TRPV3, TRPV4, TRPC4, and TRPM8 as the potential molecular targets for 3’-O-methylorobol.

### 2.6. 3’-O-Methylorobol Ameliorates Itch in Mouse Model of Histamine-Dependent Itch Induced by Compound 48/80

**Figure 6:**
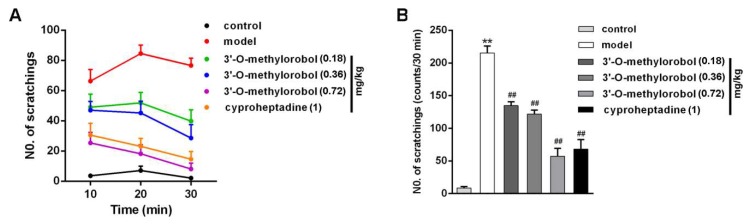
Influence of 3’-O-methylorobol on histamine-dependent itching in a mouse model induced by compound 48/80. (**A**) Effect of 3’-O-methylorobol on compound 48/80-induced spontaneous scratching in ICR mice. The number of scratches was counted for 30 min after compound 48/80 injection. (**B**) Quantification of the anti-itching effect of 3’-O-methylorobol. Data points are shown as the mean ± SEM; *n*= 7–11. ** *p* < 0.01, model group vs. control group; and ^##^ *p* < 0.01, drug vs. model group.

The hypodermic injection of compound 48/80 caused a histamine-dependent itch scratching response in mice [19]. The scratching number was 215 ± 11 times for compound 48/80 injection, and the number was 13 ± 4 times for the sham group within 30 min. Pretreatment with 3’-O-methylorobol for 30 min attenuated the scratching response (Figure 6A). Compared with the model, 3’-O-methylorobol effectively reduced spontaneous scratching bouts by 33.45% (*n* = 7, *p* < 0.01), 38.45% (*n* = 7, *p* < 0.01), and 70.46% (*n* = 7, *p* < 0.01) at doses of 0.18, 0.36, and 0.72 mg/kg, respectively (Figure 6B). As a positive control, cyproheptadine (an H_1_ receptor antagonist) also reduced the scratching number by 66.63%. Therefore, 3’-O-methylorobol at a dose of 0.72 mg/kg had a comparable efficacy against histamine-dependent itching to cyproheptadine.

### 2.7. Effect of 3’-O-Methylorobol on Itch-Evoked C-fos Expression

**Figure 7:**
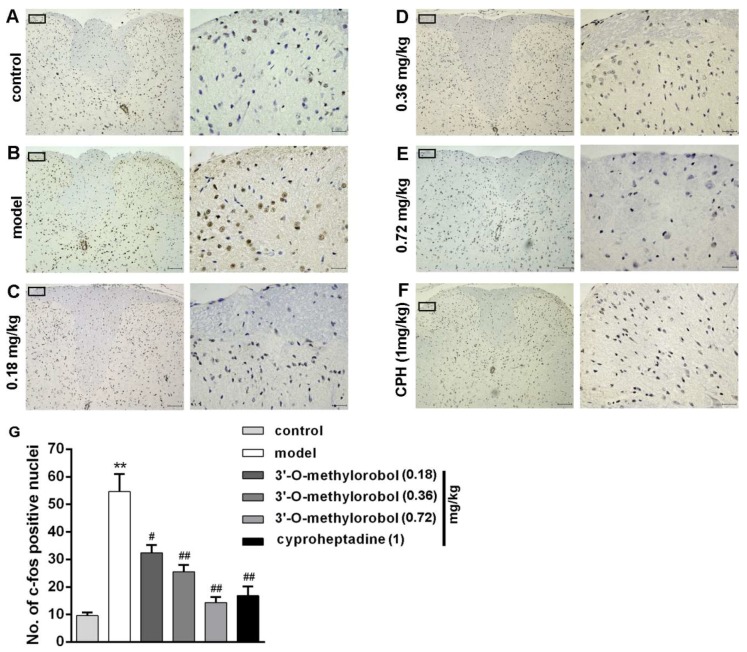
Effect of 3’-O-methylorobol on compound 48/80-evoked *c-fos* expression. Representative images of *c-fos* expression in the control group (**A**), model group pretreated with compound 48/80 (100 μg/mouse) (**B**), 3’-O-methylorobol group at 0.18 mg/kg (**C**), 0.36 mg/kg (**D**), 0.72 mg/kg (**E**), and cyproheptadine group (1 mg/kg) (**F**). Quantification of the 3’-O-methylorobol suppression of *c-fos* expression (**G**). Data points are represented as the mean ± SEM (*n* = 7–11). ** *p* < 0.01, model group vs. control group; ^#^ *p* < 0.05, drug vs. model group; and ^##^ *p* < 0.01, drug vs. model group. Scale bars, (**A**–**F**): left 100 μm; right 25 μm.

*C-fos* positive nuclei (brown-stained cell nuclei) were detected on the superficial laminas of the lateral dorsal horns of the cervical spinal cords in the compound 48/80-induced histamine-dependent itch model. Compared with the control group, the expression levels of *c-fos* in the cervical spinal segments were dramatically enhanced by pruritic agent compound 48/80 (Figure 7A,B). The administration of 3’-O-methylorobol suppressed the *c-fos* expression of the neurons, and this inhibition was 28.04% for 0.18 mg/kg, 43.39% for 0.36 mg/kg, and 68.19% for 0.72 mg/kg (Figure 7C–E). As a positive control, the administration of cyproheptadine (1 mg/kg) suppressed the *c-fos* expression of the neurons by 62.64% (Figure 7F).

## 3. Discussion

Itch-related ion channels are the key regulators in itch progression [20]. Recent evidence has indicated that the TTX-S VGSC subtype Nav1.7 in primary sensory neurons plays a significant role in mediating itching [15,17,18]. The discovery of novel gating modifiers of Nav1.7 should hold significant potential for effective treatment of itching. 3’-O-methylorobol was previously isolated from numerous medicinal plants, including *Alhagi maurorum* (antiasthmatic, antirheumatic, and demulcent), *Flemingia philippinensis* (antioxidative and anti-inflammatory), *Dalbergia parviflora* (expectorant, cardiotonic, and antipyretic), *Millettia nitida* var. hirsutissima (the promotion of blood circulation and the relief of stasis), and *Erythrina eriotriocha* Harms [21,22,23,24,25,26,27]. However, the bioactivity of 3’-O-methylorobol has rarely been reported, with the exception of it increasing alkaline phosphatase activity (an osteoblast differentiation marker) [28]. In the present study, we demonstrated that 3’-O-methylorobol is a potent Nav1.7 inhibitor.

The inhibitory effect of 3’-O-methylorobol on Nav1.7 was first examined in heterologous expressing system. 3’-O-methylorobol potently suppressed the Nav1.7 currents in CHO cells with an IC_50_ value of 3.46 μM. As a threshold channel producing Aps firing in the peripheral nociceptors, Nav1.7 is highly expressed and is the major contributor of TTX-S VGSC subtypes in large diameter DRG neurons, while TTX-R VGSCs are highly expressed in small diameter DRG neurons [29]. 3’-O-methylorobol inhibited the TTX-S currents in large diameter DRG neurons with an IC_50_ value of 6.6 μM but moderately inhibited the TTX-R currents in the small diameter DRG neurons with a maximal inhibition of 43% at a concentration of 30 μM. Nav1.6 and Nav1.8 are the predominant Na^+^ channels expressing in DRG neurons. 3’-O-methylorobol showed a lesser inhibition on the currents of Nav1.6 and Nav1.8 over Nav1.7. Recently, except for the classical histamine H_1_ receptor, many studies have indicated that TRP channels, including TRPV1, TRPV3, TRPV4, TRPC4 and TRPM8, are crucially involved in histaminergic itch generation under both physiological and pathological conditions [7,8,9,10,11,12,13]. The evaluation of the effects of 3’-O-methylorobol on the H_1_ receptor and these itch-related TRP ion channels showed that there were no obvious activities on these targets. These data together suggest that 3’-O-methylorobol is a non-selective inhibitor of Nav1.7 with some preference for Nav1.7 over Nav1.6 and Nav1.8. These data also suggest 3’-O-methylorobol was inactive in an array of histamine-dependent itch-related receptor and ion channels.

VGSCs initiate the rising phase of action potentials, therefore controlling the excitability of neurons throughout the nervous system. Based on the crucial roles of VGSCs, many drugs and toxins have been found to target VGSCs. These drugs and toxins distinctly bind to at least seven binding sites (sites 1–7), causing different electrophysiological effects [30]. Pore-blocking toxins block Na^+^ conductance by binding to site 1 (such as TTX and μ-conotoxins), and extracellular-voltage sensor toxins shift the activation voltage by biding to site 4 (such as β-scorpion toxins and spider toxins), which can result in the inhibition of Na^+^ currents [31]. Macroscopically, 3’-O-methylorobol suppressed the peak currents of Nav1.7, similar to pore-occluding toxins. Kinetically, 3’-O-methylorobol not only produced a depolarization shift of the activation voltage but also caused a hyperpolarization shift of inactivation, and the inhibition was reversible. These features are consistent with β-spider toxins and δ-atracotoxins, which modulate the gating kinetics of VGSCs [32,33,34]. 3’-O-methylorobol has a more potent inhibition in an inactivated state (−60 mV clamped voltage) than that in a resting state (−120 mV clamped voltage). The repriming time of Nav1.7 current recovery was prolonged, confirming that the inhibition of Nav1.7 currents by 3’-O-methylorobol was state-dependent. In general, site 4 modulators bind to the voltage sensor of domain II, thus affecting its activation. Whether 3’-O-methylorobol interacts with the S1–S2 linker and the S3–S4 linker of the domain II of Nav1.7 requires further exploration.

In the past decade, an increasing number of inhibitors of itch-related ion channels have been reported for itch relief. Osthole, a TRPV1 inhibitor, potently inhibits the compound 48/80-induced scratching behavior [35]. Forsythoside B selectively blocks overactive TRPV3, attenuating acute itching [12]. Menthol has been used as an itch treatment for a long time, and it has been proven that the inhibition of histaminergic itching requires the modulation of the TRPM8 channel [8]. Here, in vivo, the efficacy of 3’-O-methylorobol on itch relief in histaminergic itching induced by compound 48/80 was evaluated, an evaluation in which the spontaneous scratching bouts of mice were remarkably reduced. *C-fos* expression in the nuclei of neurons can be rapidly enhanced following pruritic agent stimulation (compound 48/80, 5-hydroxytryptamine) in the spinal dorsal horn, and it has therefore been used as a marker in pruriceptive processing [36,37,38]. Consistent with the efficacy in reducing the scratching bouts, 3’-O-methylorobol suppressed *c-fos* expression to a degree that was comparable to that of cyproheptadine. The results indicated that plant-derived inhibitors of itch-related ion channels have significant potential for the treatment of itching.

In summary, Nav1.7 has been proven to be a key and challenging target for the discovery and development of itch therapeutics. In the present work, we identified 3’-O-methylorobol as a new Nav1.7 inhibitor. 3’-O-methylorobol inhibited Nav1.7 currents by shifting the activation to the depolarization direction and the inactivation to the hyperpolarization direction, whereas it was inactive in the histamine H_1_ receptor and other itch-related ion channels such as TRPV1, TRPV3, TRPV4, TRPC4 and TRPM8. More importantly, 3’-O-methylorobol displayed a comparable anti-itch efficacy to cyproheptadine in the histaminergic itch mouse model. Taken together, our findings indicate that small molecule inhibiting Nav1.7 may represent a useful strategy to develop novel therapeutics for itching and that 3’-O-methylorobol may represent a potential lead compound to develop the treatment of histamine-dependent itching.

## 4. Materials and Methods

### 4.1. Materials and Animals

3’-O-methylorobol was purified as described previously with a purity ≥ 95%, as determined by high-performance liquid chromatography [39]. G-418, penicillin, streptomycin, 0.05% trypsin-EDTA, DMEM was obtained from Life Technology (Grand Island, NY, USA). FBS, HEPES, poly-l-lysine (PLL), poly-d-lysine (PDL, molecular weight > 300,000), and all inorganic salts were obtained from Sigma-Aldrich (St. Louis, MO, USA).

Male ICR mice (aged 7–9 weeks and weighing 25–30 g) were obtained from the Experimental Animal Center of Yangzhou University (Yangzhou, China). Animal protocols were approved by the Experimentation Ethics Committee of China Pharmaceutical University. The approval number and approval date are 20160319 and Mar.13, 2016, respectively. All efforts obeyed the general rule to reduce animal suffering and numbers.

### 4.2. Cultures of CHO, HEK293, and ND7/23 Cells

CHO cells stably expressing hNav1.6 and hNav1.7 (generously gifted from Dr. Christopher Lossin, University of California, Davis, CA, USA), HEK293 cells stably expressing hTRPV1, mTRPV3, hTRPV4, mTRPC4, and hTRPM8 (generously gifted from Dr. Michael Xi. Zhu, University of Texas Health Science Center, Houston, TX, USA), CHO cells stably expressing histamine H_1_ receptor, and ND7/23 cells transiently expressing mNav1.8 were cultured in DMEM containing 10% FBS, 100 units/mL of penicillin, 0.1 mg/mL of streptomycin and 400 μg/mL of G-418 [40,41]. All cells were cultured on PDL-precoated T-25 flasks under standard culture conditions (5% CO_2_ and 37 °C).

### 4.3. Primary Cultures of Dorsal Root Ganglion Neurons

DRG neurons were acutely dissociated from adult ICR mice according to a modified method [42]. Briefly, DRG neurons were treated with serum free-DMEM (protease, 5 mg/mL; collagenase, 2 mg/mL) for 20 min. The dissociated neurons were plated into 35-mm diameter dishes (50 µg/mL PLL-coated) and cultured in DMEM supplemented with 10% FBS. For recording TTX-S Na^+^ currents, the large DRG neurons (diameter > 45 μm) were selected. For recording TTX-R Na^+^ currents, the small DRG neurons (diameter < 20 μm) were selected. TTX (1 μM) was incubated for the inhibition of TTX-S Na^+^ currents from the mixture currents to obtain TTX-R Na^+^ currents.

### 4.4. Whole-Cell Voltage-Clamp Electrophysiology

VGSC currents were recorded with a whole-cell voltage-clamp using an EPC-10 amplifier (HEKA, Germany), as described before [43]. For Na^+^ current recordings, cells were bathed in (in mM) 140 NaCl, 3 KCl, 1 CaCl_2_, 1 MgCl_2_, and 10 HEPES (pH 7.3, adjusted with NaOH). Pipettes (1–3 MΩ) were filled with (in mM) 140 CsF, 10 NaCl, 1 EGTA and 10 HEPES (pH 7.3, adjusted with CsOH). For current-clamp recording in the DRG neurons, pipettes (2.0–4.0 MΩ) were filled with (in mM) 140 KCl, 5 MgCl_2_, 2.5 CaCl_2_, 5 EGTA, 4 ATP, 0.3 GTP, and 10 HEPES (pH 7.3, adjusted with KOH) as well as bathing solution containing (in mM) 140 NaCl, 1 MgCl_2_, 5 KCl, 2 CaCl_2_, 10 HEPES, and 10 glucose (pH 7.3, adjusted with NaOH). The osmolarity of all solutions was 300–310 mOsm. For the collection and analysis of the experimental data, PatchMaster (HEKA Electronics, Lambrecht, Germany) and GraphPad Prism (version 5.0, San Diego, California, USA) were used. The concentration–response curves of 3’-O-methylorobol were fitted to the Hill equation: I_norm_ = C + A/(1 + ((3’-O-methylorobol)/IC_50_)*^p^*), where I_norm_ is the normalized peak current, IC_50_ is the half-maximal inhibitory concentration, and *p* is the slope factor. The steady-state inactivation curve was fitted to the Boltzmann equation: y = 1/(1 + exp(V_1/2_ − V)/k), where V_1/2_ is the membrane potential at half-maximal inactivation, V is the membrane voltage of the conditioning step, and k is the slope factor. The τ_on_ and τ_off_ values were fitted using the single exponential fits equations I(t) = a_0_ + a_1_(1 − exp(−t/τ_on_)) and I(t) = a_0_ + a_1_ exp(−t/τ_off_), respectively.

### 4.5. Intracellular Ca^2+^ Concentration Determination

CHO cells stably expressing the histamine hH_1_ receptor and HEK293 cells stably expressing hTRPV1, mTRPV3, hTRPV4, mTRPC4, and hTRPM8 were used for the intracellular Ca^2+^ concentration measurements, as described previously [44]. Generally, after incubation with Calcium 6 (in Locke’s buffer) for 2 h or 4 μM of Fluo-4 (in Locke’s buffer) for 45 min, the cells were washed five times and transferred to a FLIPR^Tetra^ (Molecular Devices, Sunnyvale, CA, USA). Cell fluorescent signals were monitored before and after compound addition by a programmable pipetting system. Data are shown as F/F_0_, where F represents the fluorescent signal at different time points and F_0_ represents the average of the basal fluorescence from the initial five data points.

### 4.6. Compound 48/80-Induced Histamine-Dependent Itch Model

The back of each mouse’s neck was shaved with electric hair clippers 24 h before the experiments. Mice were placed in individual polyvinyl chambers (20 × 15 × 20 cm) and acclimated for 1 h. After the intrathecal administration of 3’-O-methylorobol or the intragastric administration of cyproheptadine for 30 min, the pruritic reagent compound 48/80 (100 μg in 50 μL) was immediately administered via hypodermic injection. The number of scratch bouts of each mouse was counted for 30 min in a 10 min interval by an observer blinded to the treatments [45].

### 4.7. C-fos Immunohistochemistry

After the administration of 3’-O-methylorobol, cyproheptadine, or compound 48/80 for 2 h, the mice were anesthetized with chloral hydrate (0.4 g/kg, intraperitoneal injection) and intracardially perfused with ice-cold 0.1 M PBS followed by 4% paraformaldehyde (in 0.1 M PBS with 0.2% picric acid). The fixed cervical spinal cords (C5–C7) were obtained and post-fixed in a 4% paraformaldehyde solution for 12 h at 4 °C for immunohistochemical staining, as previously described [36]. The tissue samples were sectioned, stained and incubated with the rabbit *c-fos* antibody at a 1:200 dilution (GB11069, Servicebio, Wuhan, China) for 12 h at 4 °C. After rinsing with PBS, the sections were incubated in biotinylated goat anti-rabbit secondary antibody for 1 h at room temperature. *C-fos*-positive nuclei were observed using a light microscope and counted at 400× magnification. The quantification of *c-fos* positive nuclei were processed by Image-Pro Plus 6.0 (Media Cybernetics, Newburyport, MA, USA).

### 4.8. Data Analysis

PatchMaster (HEKA Elektronik, Germany), and GraphPad Prism 5 (GraphPad Software, San Diego, CA, USA) software were used for data analysis. Data points are shown as the mean ± SEM. *n* is the number of the independent experimental cells or mice. Statistical significance was examined by *t*-test (two groups) or one-way ANOVA (multiple groups). *p* values below 0.05 were considered statistically significant.

## Supplementary Materials

Supplementary materials can be found at https://www.mdpi.com/1422-0067/20/23/6058/s1.

## Abbreviations

APs	Action potentials
CI	Confidence interval
DRG	Dorsal root ganglion
FLIPR	Fluorescence imaging plate reader
IC_50_	Half-maximal inhibitory concentration
I–V	Current–voltage
PDL	Poly-d-lysine
PLL	Poly-l-lysine
TRP	Transient receptor potential
TTX-R	Tetrodotoxin-resistant
TTX-S	Tetrodotoxin-sensitive
V_1/2_	Half-maximal voltage
VGSC	Voltage-gated sodium channel
