# Supplementary material for: 3’-O-Methylorobol Inhibits the Voltage-Gated Sodium Channel Nav1.7 with Anti-Itch Efficacy in A Histamine-Dependent Itch Mouse Model
Source: Int J Mol Sci. 2019 Dec 1;20(23):6058. doi: 10.3390/ijms20236058 (PMC6928743; doi:10.3390/ijms20236058)
Supplement: Supplementary file 1 [file ijms-20-06058-s001.pdf]

International Journal of
Molecular Sciences

Supplementary Information

Article

3′-O-Methylorobol Inhibits the Voltage-Gated Sodium Channel Nav1.7 with Anti-Itch Efficacy in A Histamine-Dependent Itch Mouse Model

Fan Zhang†, Ying Wu†, Shuwen Xue, Shuangyan Wang, Chunlei Zhang* and Zhengyu Cao*

Contains the following:

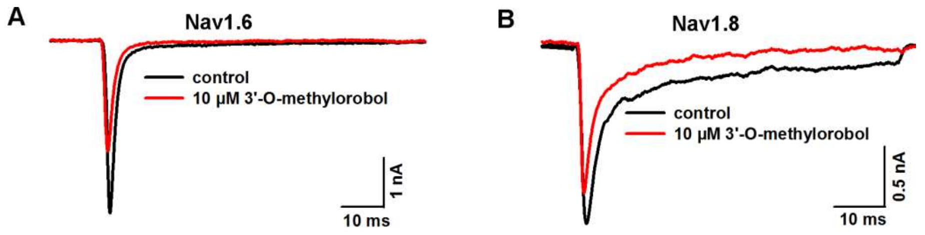

Figure S1. Effects of 3′-O-methylorobol on Nav1.6 current expressed in CHO cells and Nav1.8 current expressed in ND7/23 cells. (A) Representative traces of Nav1.6 currents before and after the applications of 3′-O-methylorobol. Nav1.6 current was triggered by a 50-ms depolarizing voltage of -20 mV from the clamped voltage of -80 mV. (B) Representative traces of Nav1.8 currents before and after the applications of 3′-O-methylorobol. Nav1.8 current was triggered by a 50-ms depolarizing voltage of -0 mV from the clamped voltage of -80 mV. n = 4-6.

© 2019 by the authors. Submitted for possible open access publication under the terms and conditions of the Creative Commons Attribution (CC BY) license (http://creativecommons.org/licenses/by/4.0/).
